# Non-endoscopic Biomarkers and Sampling for Diagnosis and Monitoring of Adult Eosinophilic Esophagitis: A Systematic Review and Pooled Analysis

**DOI:** 10.7759/cureus.91057

**Published:** 2025-08-26

**Authors:** Hao The Nguyen, Arman Vaghefi, Shania Darville, Paul Pardo, Michelle Kem Hor

**Affiliations:** 1 Internal Medicine, College of Osteopathic Medicine, Rocky Vista University, Parker, USA; 2 Internal Medicine, University of New Mexico, Albuquerque, USA; 3 Medical Sciences, University of South Florida, Tampa, USA; 4 College of Arts and Sciences, University of South Florida, Tampa, USA; 5 Gastroenterology, UCHealth Memorial Central Hospital, Colorado Springs, USA

**Keywords:** biomarker assessment, clinical diagnostic value, endoscopy, eosinophilic esophagitis (eoe), serum biomarker

## Abstract

Eosinophilic esophagitis (EoE) typically requires endoscopic biopsy for diagnosis and disease monitoring, but less invasive tools may offer clinical utility. This systematic review evaluated the diagnostic performance and treatment responsiveness of non-endoscopic biomarkers and sampling techniques in adult EoE. Sixteen studies met the inclusion criteria. Diagnostic biomarkers such as eosinophil-derived neurotoxin (EDN), major basic protein (MBP), and eosinophilic cationic protein (ECP) showed inconsistent performance across studies. Absolute eosinophil count (AEC) was significantly elevated in active EoE, though reported in only one study. Minimally invasive sampling tools, including cytosponge and liquid-based cytology (LBC), demonstrated moderate sensitivity (70-75%) and specificity (56-86%) for the diagnosis of EoE. For monitoring treatment response, pooled analysis revealed significant reductions in AEC, ECP, and EDN following therapy, while MBP, eotaxin-3, IL-5, and IL-13 showed no meaningful change. Risk of bias was moderate, largely due to variability in biomarker thresholds and lack of treatment stratification. These findings suggest non-endoscopic tools may complement standard histologic evaluation but require prospective validation in larger cohorts before routine clinical use.

## Introduction and background

Eosinophilic esophagitis (EoE) is a chronic, immune-mediated disease of the esophagus that presents with symptoms including dysphagia, heartburn, regurgitation, and recurrent food impaction [1]. This condition has been continually increasing in prevalence, and EoE-associated healthcare costs in the United States have been estimated to be upward of $1.3 billion [2,3]. The gold standard for both diagnosing and monitoring EoE continues to be endoscopic biopsy, requiring at least 15 eosinophils per high-power field (eos/hpf) on light microscopy [1]. Frequent endoscopy is costly and burdensome to both patients and healthcare systems, highlighting the need for advancement in management.

There is growing interest in the development of non-endoscopic tools for diagnosing and monitoring EoE, but their clinical performance and utility have yet to be clearly defined. Prior systematic reviews, including those published through 2018, have highlighted substantial heterogeneity across studies and confounding from coexisting atopic conditions as key barriers to interpretation [4,5]. There has been a continued growing interest in developing less invasive techniques to diagnose and monitor EoE since these reviews were published. This systematic review aims to update and synthesize more recent evidence on non-endoscopic biomarkers and sampling techniques in adult EoE, with a focus on evaluating their diagnostic accuracy and responsiveness to treatment.

## Review

Methods

Study Design and Search Strategy

This systematic review was conducted in accordance with the Preferred Reporting Items for Systematic Reviews and Meta-Analyses (PRISMA) 2020 guidelines. The protocol for this review was prospectively registered in the International Prospective Register of Systematic Reviews (PROSPERO; registration ID: CRD420251040649), which initially included both adult and pediatric populations in its scope. However, this manuscript presents a focused analysis restricted to adult patients (≥18 years). This restriction to adult data was applied prior to data extraction and analysis for this manuscript and is explicitly disclosed to preserve methodological transparency. All other components of the protocol were followed as registered.

A systematic search was performed in PubMed, Embase, and Cochrane Central utilizing a carefully curated combination of MeSH terms and the following key words: "eosinophilic esophagitis," "oeosinophilic esophagitis," "EoE," "biological markers," "biomarkers," "non-invasive," "non-endoscopic," "Cytosponge," and "minimally invasive." The search was modified to match the requirements of each database. 

Eligibility Criteria 

Studies were included if they enrolled adults with biopsy-confirmed EoE, evaluated non-endoscopic biomarkers or sampling tools, and reported diagnostic or monitoring outcomes against a reference standard. Eligible designs included randomized trials, cohort, cross-sectional, or case-control studies published in English between January 1, 2014, and May 30, 2025. For the biomarker sub-analysis, studies were required to report paired pre- and post-treatment biomarker values with sufficient data to estimate variance. Sixteen studies met the inclusion criteria for the qualitative synthesis, and 10 were eligible for the treatment response sub-analysis.

Study Selection and Data Extraction

Two reviewers independently extracted data on study design, patient population, biomarkers, sample size, diagnostic metrics, treatment modality, and pre-/post-treatment values. Risk of bias was evaluated using the QUADAS-2 tool, which assesses four domains: patient selection, index test, reference standard, and flow and timing. Two reviewers independently applied the tool to each included study, with disagreements resolved by consensus. Assessments were recorded for each domain and used to inform qualitative interpretation of the findings. Discrepancies were resolved with a third reviewer.

Data Synthesis

For biomarkers with ≥2 studies reporting pre- and post-treatment values, mean changes were pooled using a fixed-effect model with sample-size weighting. A fixed-effect approach was chosen due to the limited number of studies per biomarker. When standard deviations were not reported, they were imputed using the method of Wan et al. [6] with an assumed correlation coefficient of r=0.5. Due to broader heterogeneity in study designs and outcome reporting, the remaining findings were summarized descriptively in tables and figures.

Results

**Figure 1:**
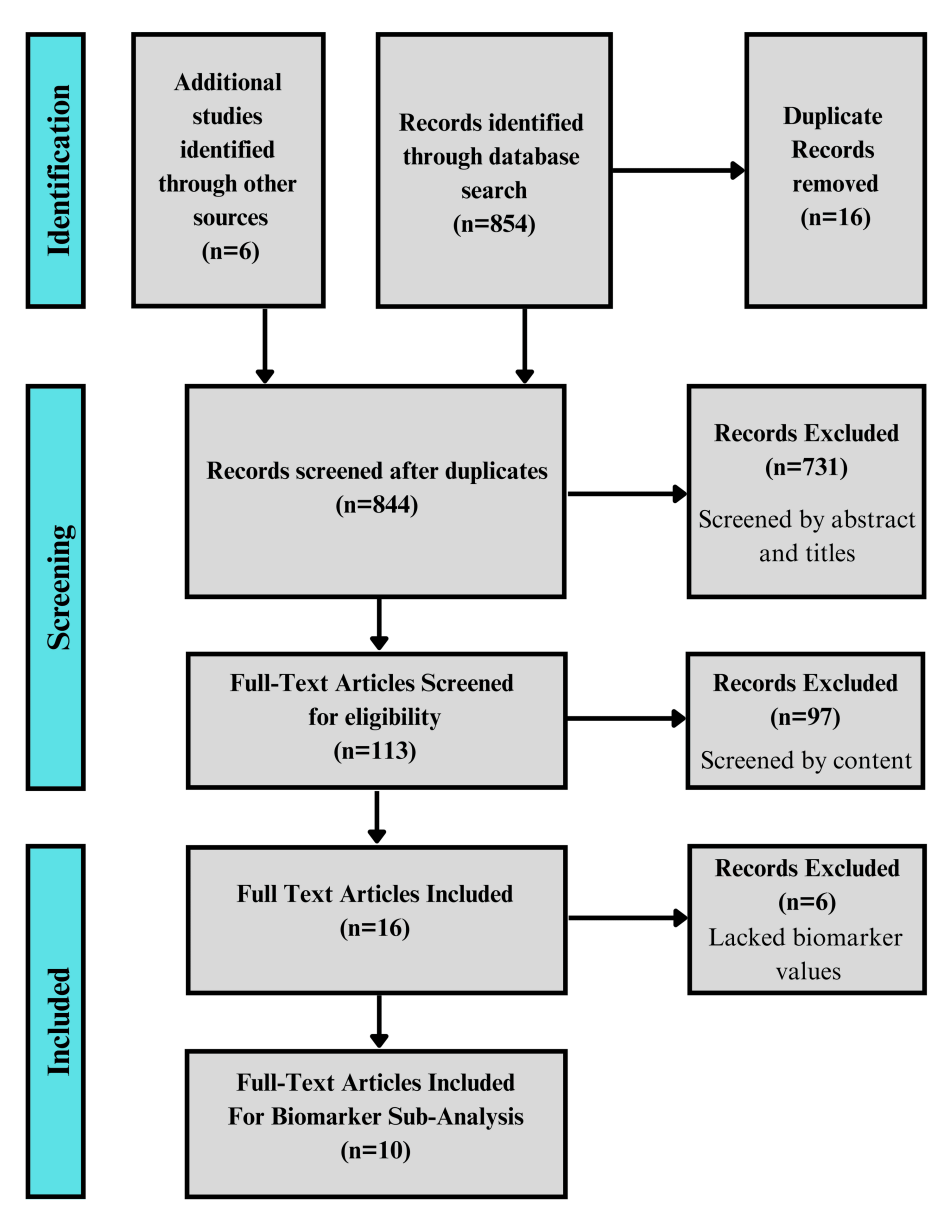
PRISMA diagram illustrating the steps of study selection for systematic review PRISMA, Preferred Reporting Items for Systematic Reviews and Meta-Analyses

**Figure 2:**
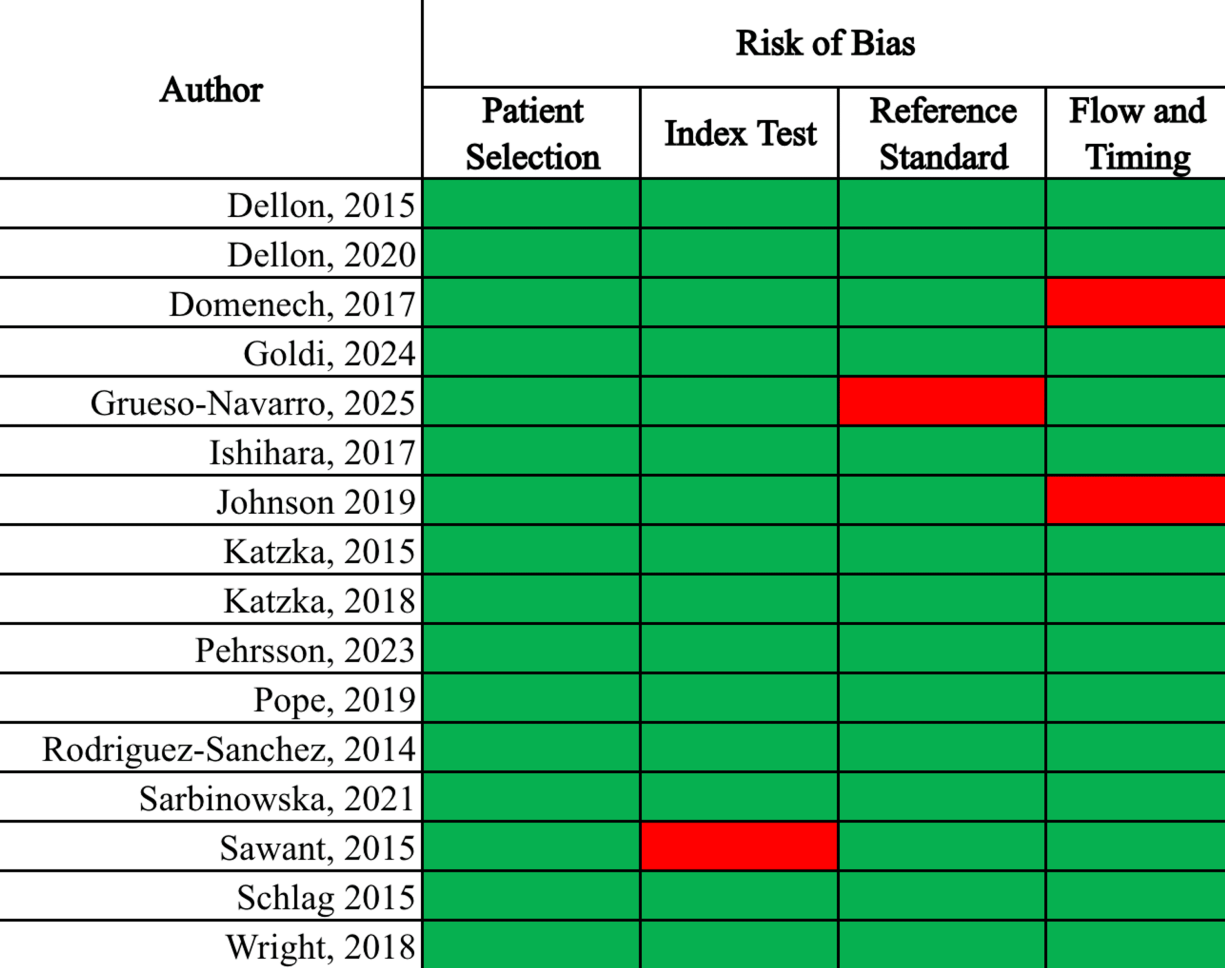
QUADAS-2 heat map illustrating the risk of bias assessment for each included study The QUADAS-2 heat map summarizes risk of bias and applicability concerns across four domains in the included studies [7-23]. Green indicates low risk or low concern, and red indicates high risk or high concern. This visual highlights methodological strengths and limitations across the evidence base.

A total of 854 records were identified through the initial database search, with an additional six studies identified through other sources. After removal of 16 duplicates, 844 records remained for title and abstract screening. Of these, 731 were excluded for not meeting the inclusion criteria. The remaining 113 full-text articles were assessed for eligibility. Ultimately, 16 studies met all inclusion criteria and were included in the qualitative synthesis. From this group, 10 studies reported paired pre- and post-treatment biomarker data and were included in the quantitative sub-analysis of treatment response. The full study selection process is illustrated in the PRISMA flow diagram (Figure 1). The risk of bias of these studies was then assessed utilizing the QUADAS-2 tool, as seen in Figure 2. 

A brief description of each of the included studies is highlighted in Table 1. From the 16 included studies, over 40 eosinophil-related biomarkers were evaluated for their diagnostic utility in adult EoE, but only a limited subset showed consistent or clinically relevant findings. Eosinophil-derived neurotoxin (EDN) was assessed in three studies (n=118), major basic protein (MBP) in four studies (n=115), and eosinophilic cationic protein (ECP) in four studies (n=58) [7-10]. Across these biomarkers, results were inconsistent when comparing active EoE to control groups. Fractional exhaled nitric oxide (FeNO), evaluated in two studies (n=88), showed no significant correlation with peak eosinophil counts per high-power field (eos/hpf) [8,11]. Absolute eosinophil count (AEC) was significantly elevated in patients with active EoE compared to controls, but this finding was limited to a single study (n=38) [8]. A complete summary of diagnostic biomarker findings is presented in Table 2 [7-15].

**Table 1:** Brief description of the studies included in this systematic review A brief description of each of the included studies [7-23]. EDN, eosinophil-derived neurotoxin; MBP, major basic protein; LBC, liquid-based cytology; AEC, absolute eosinophil count; ECP, eosinophilic cationic protein; FeNO, fractionated exhaled nitric oxide

Study	Year	Biomarker(s) explored	Alternative technique explored	Collection
Dellon et al.	2015	IL-4, IL-5, IL-6, IL-9, IL-13, TGF-α, TGF-β, TNF-α, eotaxin-1, -2, and -3, TSLP, MBP, and EDN	NA	Serum
Dellon et al.	2020	Eotaxin-3, tryptase, and MBP	NA	Serum
Domenech et al.	2017	ECP	NA	Serum
Goldi et al.	2024	FeNO, AEC, EDN, ECP, MBP-1, thymus, and activation of regulated chemokine	NA	Serum
Grueso-Navarro et al.	2025	Leu_Comb_5, Ser_Comb_22, hsa-miR-10b-5p, hsa-miR-125a-5p, hsa-let-7d-5p, hsa-miR-224-5p, hsa-miR-221-3p, hsa-miR-191-5p, hsa-miR-15a-5p, hsa-let-7d-5p, hsa-miR-30a-3p, hsa-miR-374-5p	NA	Serum
Ishihara et al.	2017	Eotaxin-3, GRO, IP-10, MCP-1, MDC, RANTES, BCA-1, CTACK, ENA-78, eotaxin-2, MIP-1δ, SDF-1, TARC, GCP-2, HCC-1, I-TAC, MIG, MIP-3β, NAP-2, SCCA1, SCCA2, and periostin	NA	Serum
Johnson et al.	2019	NA	FeNO	NA
Katzka et al.	2015	NA	Cytosponge	NA
Katzka et al.	2018	NA	Cytosponge	NA
Pehrsson et al.	2023	PRO-C3, PC3X, C3M, CTX-III, PRO-C4, C4M, PRO-C5, PRO-C6, C6M,VICM, VIM, and CPa9-HNE	NA	Serum
Pope et al.	2019	Esophageal IgG4	NA	Serum
Rodriguez-Sanchez et al.	2014	LBC aspirate samples	NA	Serum
Sarbinowska et al.	2021	IL-5, IL-13, transforming growth factor β1, MBP, and eotaxin 3	NA	Serum
Sawant et al.	2015	MIRNA-21 (serum) and MiR-22 (serum)	NA	Serum
Schlag et al.	2015	Absolute eosinophil count, CCL-17, CCL-18, CCL-26, eosinophil-cationic-protein, and mast cell tryptase	NA	Serum
Wright et al.	2018	Eosinophil cationic protein, EDN, and eosinophil MBP	NA	Serum

**Table 2:** Key findings and limitations of the literature search Findings and limitations were synthesized from the included studies [7-23]. AEC, absolute eosinophil count; EDN, eosinophil-derived neurotoxin; MPB, major basic protein; ECP, eosinophilic cationic protein; FeNO, fractionated exhaled nitric oxide; hpf, high-powered field

Biomarker	Studies/n	Descriptive key findings	Main limitations
AEC	2/107	Significantly higher in active EoE vs. control groups (n=38) and significant decrease post-treatment compared to baseline (n=69). Histologic remission showed a sensitivity of 88% and specificity of 56% at an AEC cut-off of 300 eosinophils/mm^3^ (n=69).	Few studies, inconsistent cut-off thresholds, and atopy confounding
EDN	3/118	Inconsistent relationship in active EoE vs. control groups (n=118). Active EoE vs. non-EoE had a sensitivity of 75.8% and specificity of 73.3% at a cutoff of 50.73 ng/mL (n=38).	Sampling heterogeneity and varied EDN cut-offs
MBP	4/225	Inconsistent relationship in active EoE vs. control groups (n=115). Elevated levels in treatment non-responders compared to responders (n=110).	Small cohorts, assay variability, and non-uniform response definitions
ECP	4/146	Inconsistent relationship in active EoE vs. control groups (n=58). Significant decrease post-treatment compared to baseline (n=108).	Variable outcome measures and limited longitudinal data
FeNO	2/88	No significant correlation with eosinophils/hpf (n=88). Diagnostic sensitivity ranged from 25% to 75.9% and specificity from 34.5% to 89% depending on the FeNO cut-off (n=88)	Few studies, breathing-technique variability, and inconsistent cut-off values between studies

**Figure 3:**
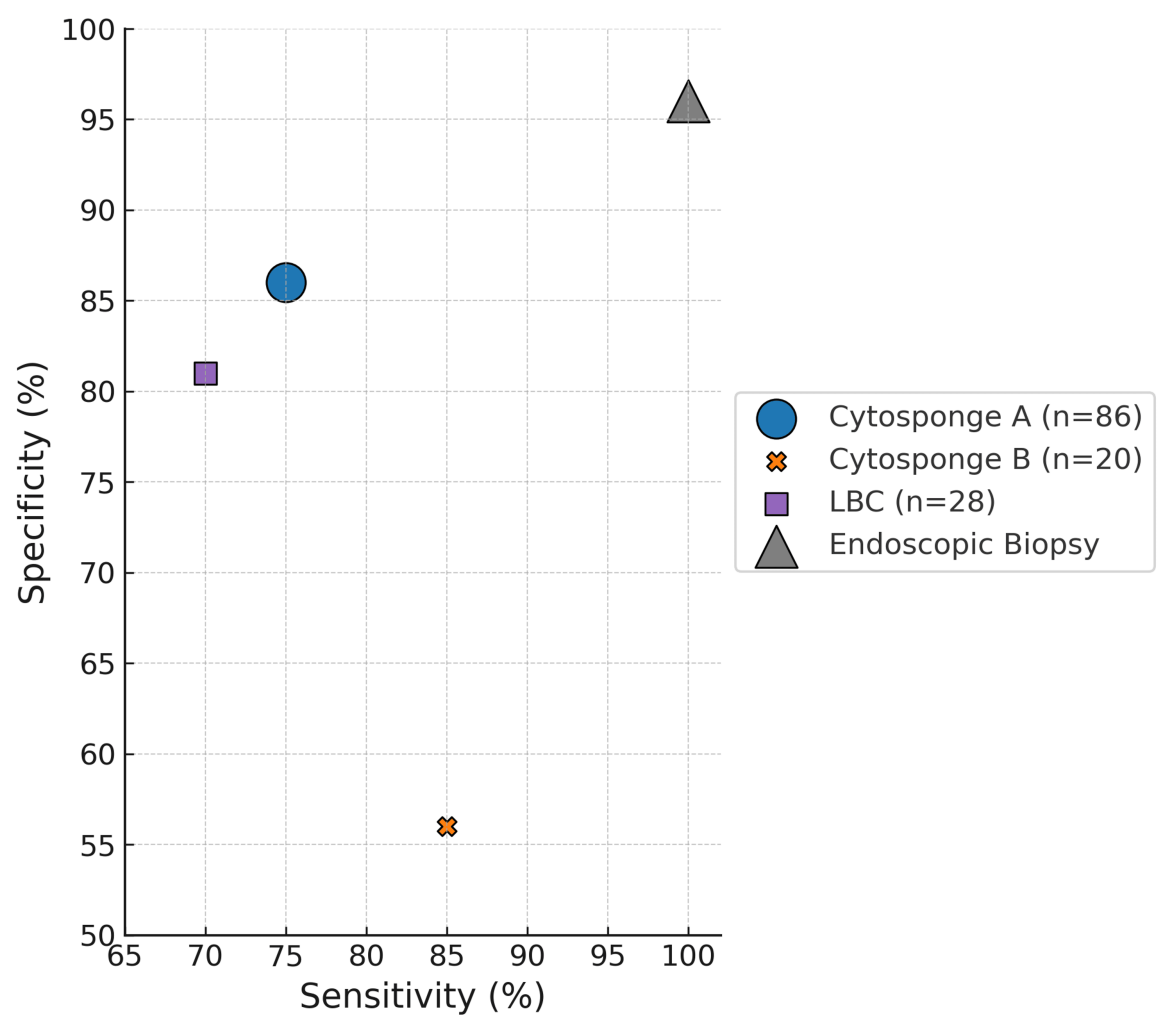
Scatterplot of diagnostic sampling tools; size of each figure is reflective of its sample size Cytosponge A and B are derived from the results of two studies investigating the collection technique [15-16], while LBC was derived from one study investigating the sampling technique [17]. LBC, liquid-based cytology

Three studies evaluated non-endoscopic sampling techniques. Cytosponge was assessed in two studies: one reported a sensitivity of 75% and specificity of 86% (n=86), while the other reported 70% sensitivity and 56% specificity (n=20) [15,16]. Liquid-based cytology (LBC), evaluated in one study, demonstrated 70% sensitivity and 81% specificity [17]. All studies defined histologic EoE using a threshold of ≥15 eos/hpf. These results are illustrated in Figure 3.

**Figure 4:**
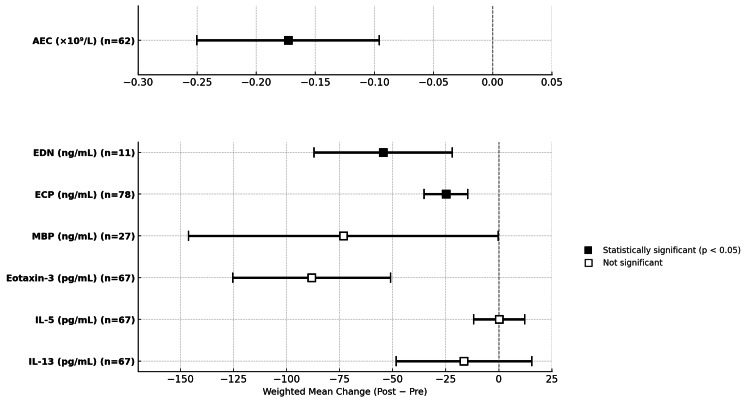
Forest plot of biomarkers and their 95% CI The figure illustrates SMDs in biomarker levels before and after treatment across included studies in adult EoE [7-13]. Filled squares indicate statistically significant changes. EoE, eosinophilic esophagitis; SMDs, standardized mean differences; EDN, eosinophil-derived neurotoxin; MBP, major basic protein; ECP, eosinophilic cationic protein; AEC, absolute eosinophil count

Several biomarkers were evaluated for their utility in monitoring treatment response, with pre- and post-treatment values pooled to calculate mean change. When standard deviations were not reported, they were computed using the Wan et al. [6] method. AEC, assessed in two studies (n=62), demonstrated a mean decrease of -0.173±0.31×10⁹/L [8,12]. EDN, reported in one study (n=11), showed a mean change of -54.44±55.1 ng/mL [8]. ECP, evaluated in three studies (n=78), decreased by -24.88±46.3 ng/mL. These mean changes reached statistical significance (p≤0.006) [8,12,13]. Other biomarkers, including MBP, eotaxin-3, IL-5, and IL-13, showed non-significant mean changes. Biomarker values were not stratified by treatment subgroups in any of these studies. Full pooled results for all biomarkers are presented in Table 3, and a forest plot showcasing their confidence intervals is illustrated in Figure 4. 

**Table 3:** Pooled mean change of biomarkers The table shows pooled mean pre- and post-treatment values for select biomarkers across studies evaluating adults with EoE. Standard deviations and p-values are reported where available. When not reported, standard deviations were imputed using the method of Wan et al. [6]. Data are synthesized from studies [7-13]. EoE, eosinophilic esophagitis; AEC, absolute eosinophil count; EDN, eosinophil-derived neurotoxin; MPB, major basic protein; ECP, eosinophilic cationic protein

Biomarker	Studies/n	Weighted mean pre-treatment	Weighted mean post-treatment	Δ mean	p-value
Serum biomarkers					
AEC (×10⁹/L)	2/n=62	0.370±0.312	0.198±0.075	-0.173±0.31	0.001, 0.0001
EDN (ng/mL)	1/n=11	67.4±55.1	11.96±28.4	-54.44±55.1	0.002
ECP (ng/mL)	3/n=78	56.5±46.3	31.62±29.6	-24.88±46.3	0.006, 0.001, 0.0016
MBP (ng/mL)	2/n=27	613.0±193.4	539.8±154.5	-73.20±193.4	0.1153, NR
Chemokine biomarkers					
Eotaxin-3 (pg/mL)	2/n=67	120.2±155.1	32.02±72.6	-88.18±155.1	0.41, NR
IL-5 (pg/mL)	2/n=67	15.48±50.1	15.65±52.9	0.17±50.1	0.81, NR
IL-13 (pg/mL)	2/n=67	54.76±133.4	38.31±89.8	-16.45±133.4	0.18, NR

Risk of bias was assessed using the QUADAS-2 tool. For studies evaluating non-endoscopic sampling techniques, the reference standard was consistent, with histologic EoE defined as >15 eos/hpf. In contrast, biomarker studies demonstrated considerable heterogeneity in thresholds used to define abnormal values, and most did not pre-specify cutoffs. Patient selection methods were generally well-described among all studies. However, biomarker response was not stratified by treatment modality in studies that featured multiple types of interventions. This limits the assessment of intervention-specific effects. Overall, the risk of bias was judged to be moderate, driven primarily by variability in index test definitions and incomplete stratification of treatment subgroups.

Discussion

*Minimally Invasive Biomarkers and Sampling Techniques*

EDN, MBP, and ECP were evaluated across multiple studies but showed inconsistent results when control groups were compared to those with active disease, likely due to differences in threshold definitions and limited patient pools. FeNO also showed no correlation with histologic eosinophil counts. AEC was significantly elevated in active EoE compared to control groups, but was only evaluated in one study. There were over 35 other biomarkers investigated among these studies, but none were found to be clinically relevant [18-23]. Among sampling techniques, Cytosponge demonstrated moderate diagnostic accuracy (70-75% sensitivity, 56-86% specificity), and LBC performed similarly (70% sensitivity, 81% specificity). 

Among the biomarkers evaluated for treatment response, AEC, ECP, and EDN showed the most promise. AEC and ECP demonstrated a significant decrease across multiple studies, indicating potential utility in monitoring disease activity. EDN was also significantly reduced post-treatment, but was only investigated in one study (n=11). Other biomarkers such as MBP, eotaxin-3, IL-5, and IL-13 exhibited no meaningful change post-treatment.

While none of the evaluated biomarkers and sampling techniques can replace histologic confirmation via endoscopic biopsy, these minimally invasive alternatives show potential to become adjunctive tools. This is especially the case for AEC, ECP, and EDN, which demonstrated statistically significant changes after patients began treatment. This suggests possible utility in monitoring disease activity. Cytosponge and LBC exhibited moderate diagnostic performance that may be valuable in patients who have contraindications to anesthesia or for whom repeated endoscopy is overly burdensome. These tools may also expand access to care in underserved settings where repeated endoscopy is not feasible. Despite promising results, these biomarkers and techniques are not yet ready for clinical use.

Comparisons to Literature

Our findings reinforce prior concerns about heterogeneity. A published review in 2012 compared results from 26 studies that investigated biomarkers for EoE between 2006 and 2012 [4]. These studies evaluated the use of EDN, AEC, IL-13, eotaxin-3, and many other biomarkers in monitoring and diagnosing EoE. Interestingly, this article described eotaxin-3 and IL-13 as the most promising biomarkers at this time. This is contrary to our findings, as these two biomarkers were found to be unremarkable in the studies that were included in our review. This may be attributed to the heterogeneity of the studies included in both articles, as this article also comments on the stark differences between methodologies and biomarkers studied.

Another systematic review was published in 2018 that included articles published from inception to June 6, 2017 [5]. Studies were included if patients met consensus criteria for EoE diagnosis, a biomarker was assessed, and the study included a control group. Unlike our study, this review included pediatric data in its evaluation and included 49 studies. This review identified AEC, ECP, chemokine receptor type 3, and eotaxin as promising biomarkers. They also highlighted many areas of improvement that future studies can make, which include prospective design, absence of an atopic control group, and timing of specimen collection. These weaknesses in study design were also prevalent in our included studies.

A recent systematic review by Noble et al. [24] examined non-invasive biomarkers for diagnosing and monitoring EoE across both pediatric and adult populations. Their study further reinforces that heterogeneity between studies is a major limiting factor within the domain of biomarker research in EoE. While comprehensive, their analysis combined studies from heterogeneous age groups without stratified synthesis, despite acknowledging important differences in EoE pathophysiology and clinical presentation between children and adults. In contrast, our review focuses exclusively on adult populations, providing a more targeted synthesis relevant to adult clinical practice. Given that biomarker profiles may differ significantly by age, this adult-specific approach adds granularity and improves applicability for guiding biomarker development and implementation in adult EoE.

Limitations

There are many other limitations to consider. The number of studies per biomarker was limited, reducing the power and generalizability of pooled estimates. Thresholds for defining abnormal biomarker levels were inconsistently reported, and many studies lacked pre-specified cutoffs. Imputing standard deviations may have introduced error, despite methodological justification. Treatment response data were not stratified by intervention type, limiting insight into biomarker specificity for particular modalities. Finally, heterogeneity in outcome definitions, assay platforms, and sampling protocols likely contributed to variation in reported performance metrics. Future research should prioritize comparisons of biomarkers and controls, stratified by treatment modality and disease severity, to evaluate their predictive value over time. 

## Conclusions

This systematic review highlights the potential role of non-endoscopic biomarkers and sampling techniques in the diagnosis and monitoring of EoE in adults. While diagnostic performance varied across biomarkers, eosinophil-derived markers such as AEC, EDN, and ECP demonstrated measurable treatment response and showed potential for monitoring disease activity. Minimally invasive sampling tools, Cytosponge and LBC, showed moderate sensitivity and specificity for the diagnosis of EoE. These non-endoscopic tools were limited by a small number of studies and patient pools, requiring prospective validation among larger cohorts before clinical utilization. Standardization of biomarker thresholds, assay methods, and treatment stratification in future studies will be essential to integrate these tools into routine clinical practice.
